# Efficacy and Safety of Adjunctive Perampanel in Young Children (7–46 Months) With Drug‐Resistant Epilepsy: A Real‐World Study

**DOI:** 10.1002/cns.70998

**Published:** 2026-06-19

**Authors:** Qiao Zeng, Xueqian Xia, Hao Zheng, Lu Yang, Li Jiang, Yue Hu

**Affiliations:** ^1^ Department of Neurology Children's Hospital of Chongqing Medical University, National Clinical Research Center for Child Health and Disorders, Ministry of Education Key Laboratory of Child Development and Disorders Chongqing China; ^2^ Chongqing Key Laboratory of Child Neurodevelopment and Cognitive Disorders Chongqing China; ^3^ Big Data Engineering Center Children's Hospital of Chongqing Medical University Chongqing China

**Keywords:** antiseizure medication, drug‐resistant epilepsy, perampanel, real‐world study, young children

## Abstract

**Background:**

To evaluate the efficacy and safety of adjunctive perampanel (PER) in young children with drug‐resistant epilepsy (DRE) aged 7–46 months, and to identify predictors of treatment response in real‐world clinical practice.

**Methods:**

We conducted a nonrandomized, open‐label, single‐arm, self‐controlled real‐world study at the Children's Hospital of Chongqing Medical University between December 2020 and August 2024. Eighty‐seven children with DRE received PER as adjunctive therapy to existing antiseizure medications (ASMs). The primary endpoint was the responder rate (≥ 50% reduction in seizure frequency) at 3, 6, 9, and 12 months. Secondary endpoints included seizure freedom, treatment retention, and treatment‐emergent adverse events (TEAEs).

**Results:**

Responder rates were 39.5%, 46.9%, 43.2%, and 44.4% at 3, 6, 9, and 12 months, respectively. Responder rates were 50.0% in Dravet syndrome, 50.0% in Lennox–Gastaut syndrome, and 34.8% in infantile epileptic spasms syndrome. Children with genetic etiologies had a 51.7% responder rate, including 60.0% in those with *SCN1A* variants. Multivariable logistic regression identified perinatal brain injury as an independent predictor of favorable response, while concomitant use of three ASMs predicted poorer outcomes. Treatment retention rates were 87.4%, 69.0%, 59.8%, and 55.2% at 3, 6, 9, and 12 months. TEAEs occurred in 23.0% of patients, most commonly somnolence (11.5%) and irritability/aggressive behavior (9.2%); 4.6% discontinued due to TEAEs.

**Conclusion:**

Adjunctive PER demonstrated clinically meaningful efficacy and a favorable safety profile in young children with DRE, supporting its potential role as a broad‐spectrum ASM in this age group.

## Graphical abstract

This real‐world study evaluates adjunctive perampanel (PER) in young children (7–46 months) with drug‐resistant epilepsy. Results show clinically meaningful efficacy, with responder rates of up to 50% in certain syndromes. PER demonstrates a favorable safety profile, supporting its role as an effective treatment option in this age group.

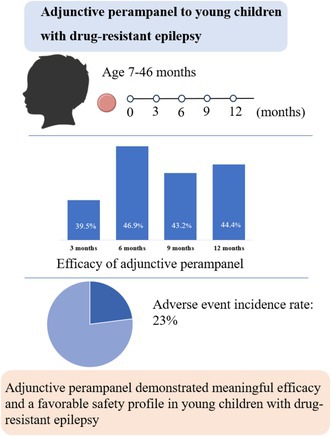

## 1 Introduction

Drug‐resistant epilepsy (DRE) is defined as the failure to achieve sustained seizure freedom after adequate trials of two tolerated, appropriately chosen, and appropriately used antiseizure medications (ASMs) [1]. Children with DRE not only experience persistent seizures but are also at high risk of adverse neurodevelopmental and cognitive outcomes. The cumulative incidence of DRE in pediatric populations is approximately 25% [2], underscoring the urgent need for effective and well‐tolerated treatments in this vulnerable age group.

Perampanel (PER) is a selective, noncompetitive antagonist of the α‐amino‐3‐hydroxy‐5‐methyl‐4‐isoxazolepropionic acid (AMPA) receptor [3]. Since its initial approval by the U.S. Food and Drug Administration in 2012, PER has been indicated as adjunctive therapy across a range of seizure types and age groups [4, 5]. In China, the National Medical Products Administration (NMPA) approved PER in 2021 as adjunctive therapy for focal seizures, with or without secondary generalization, in children aged 4 years and older [6]. Emerging evidence suggests that PER may also be effective in children younger than 4 years, with clinical use reported in infants as young as 4 months [7, 8, 9, 10]. A recent expert consensus in China further recommended PER for diverse pediatric epilepsy syndromes, including Dravet syndrome (DS) and Lennox–Gastaut syndrome (LGS) [11]. These observations raise the possibility that PER may represent an important therapeutic option for very young children with DRE, but robust real‐world evidence in this population remains limited.

We conducted a real‐world study to evaluate the efficacy, safety, and retention of PER as adjunctive therapy in children aged 7–46 months with DRE at the Children's Hospital of Chongqing Medical University between December 2020 and August 2024, and to investigate factors associated with treatment response.

## 2 Materials and Methods

### 2.1 Study Population

We conducted a nonrandomized, open‐label, single‐arm, self‐controlled real‐world study at the Children's Hospital of Chongqing Medical University between December 6, 2020 and August 10, 2024. Eligible participants were children aged 1 month to 4 years who met the 2017 International League Against Epilepsy (ILAE) diagnostic criteria for epilepsy and DRE [1, 12]. The final cohort included in the safety analysis comprised 87 children aged 7–46 months.

The study protocol was reviewed and approved by the Ethics Committee of Children's Hospital of Chongqing Medical University (approval No. 346). Written informed consent was obtained from the legal guardians of all participants prior to enrollment. Investigators explicitly disclosed the off‐label use of PER in patients outside NMPA‐approved indications, detailing potential benefits and risks, and obtained consent for such use. Participants exhibiting poor treatment adherence or whose medical histories could not be reliably confirmed were excluded from the study.

### 2.2 Study Design

PER (Eisai Co. Ltd., Kawashima Factory; registration No. H20190053) was administered orally once daily at bedtime as adjunctive therapy, without altering or replacing concomitant ASMs. The starting dose was weight‐based: 0.5 mg/day for children weighing < 20 kg and 1 mg/day for those weighing 20–30 kg. Dose titration proceeded in increments of one starting unit every 1–2 weeks, guided by clinical response and tolerability. Maintenance dosing was individualized.

Baseline seizure frequency was defined as the mean monthly frequency during the 3 months prior to PER initiation. Seizure reduction rate was calculated as (baseline frequency − post‐treatment frequency)/baseline frequency × 100%. Treatment response was defined as a ≥ 50% reduction in seizure frequency relative to baseline [13], and seizure worsening as a > 25% increase [14].

**FIGURE 1:**
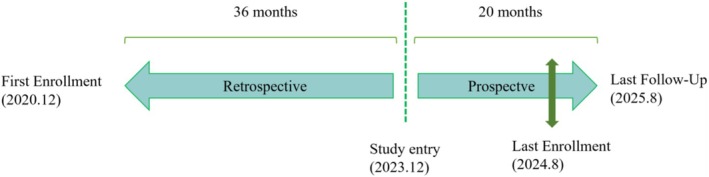
Study design of adjunctive perampanel in young children with drug‐resistant epilepsy. The study included a 36 month retrospective data‐collection phase and a 20 month prospective follow‐up phase.

The study comprised a retrospective data‐collection phase of 36 months and a prospective follow‐up phase of 20 months, yielding a total observation period of 56 months (Figure 1). The primary endpoint was the responder rate, defined as a ≥ 50% reduction from baseline in monthly seizure frequency, assessed at 3, 6, 9, and 12 months after perampanel initiation. Secondary endpoints included seizure‐freedom rate across the entire study period, treatment retention rate, and incidence of treatment‐emergent adverse events (TEAEs). Patients who initiated additional therapies during follow‐up, including ketogenic diet, epilepsy surgery, or vagus nerve stimulation, or who modified their concomitant ASM regimens were excluded from efficacy analyses. However, these patients remained eligible for safety evaluation to capture the full spectrum of TEAEs.

### 2.3 Statistical Analysis

All analyses were performed using R software (version 4.5.1). Continuous variables were expressed as medians with interquartile ranges (IQRs) and categorical variables as counts with percentages. Normality was assessed, and normally distributed variables were compared using independent‐samples *t*‐tests.

Treatment retention was estimated by the Kaplan–Meier method. Missing values considered missing at random were imputed using multiple imputation (random forest algorithm for continuous variables; multinomial logistic regression for categorical variables). Censored data due to discontinuation, regimen change, or loss to follow‐up were regarded as missing not at random and addressed using the last observation carried forward (LOCF) method. Robustness of this approach was tested using a mixed model for repeated measures (MMRM) with a Toeplitz covariance structure.

Univariable logistic regression was used to identify baseline predictors of treatment response (≥ 50% seizure reduction). Variables at *p* < 0.05 in univariable analyses were entered into a multivariable logistic regression model. A two‐sided *p* < 0.05 was considered statistically significant.

## 3 Results

### 3.1 Baseline Characteristics

**FIGURE 2:**
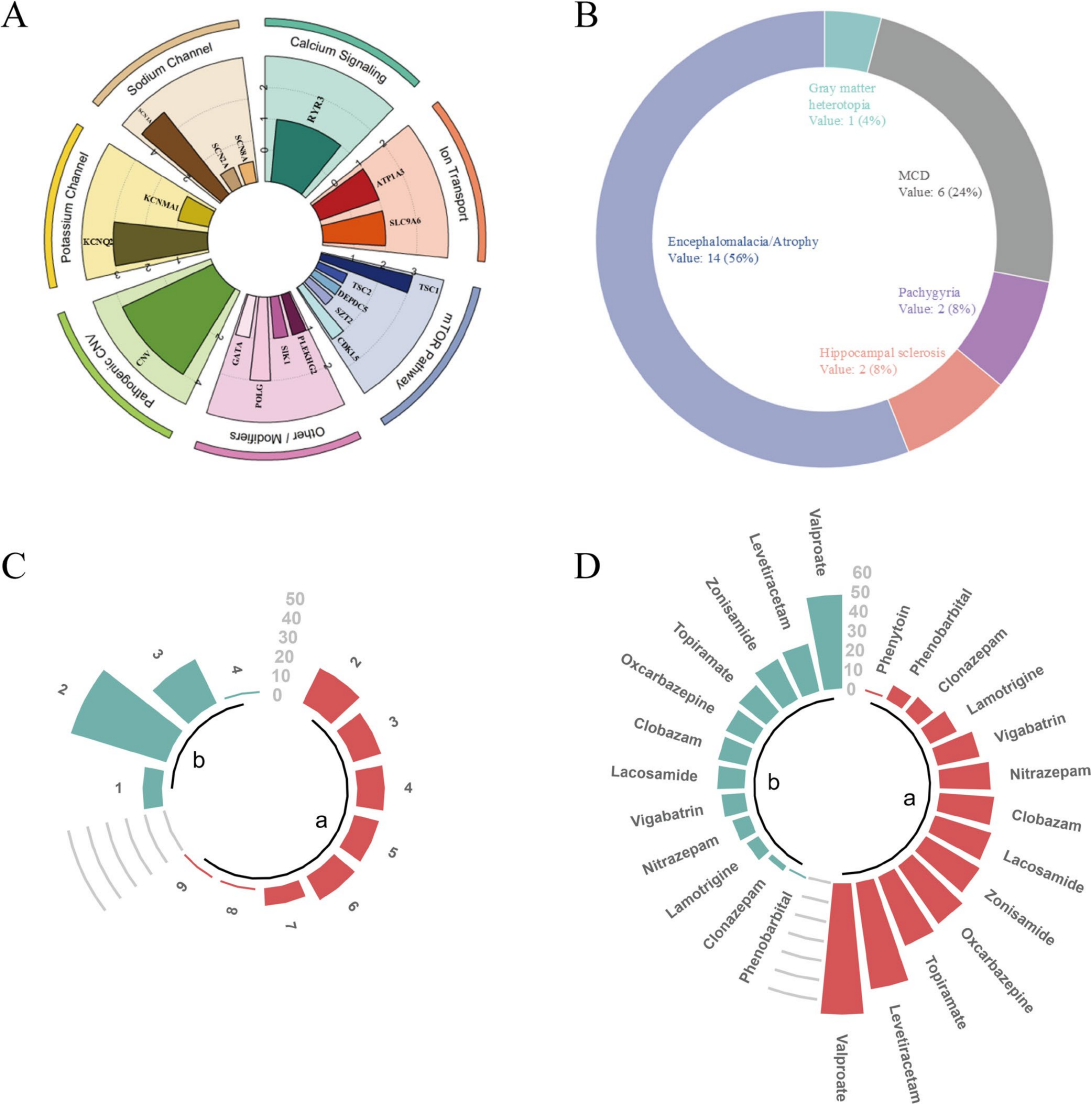
Baseline clinical characteristics of 87 young children with drug‐resistant epilepsy treated with adjunctive perampanel. (A) Genetic etiologies. (B) Structural etiologies. (C) Number of previously used ASMs (a) and number of concomitant ASMs at baseline (b). (D) Types of previously used ASMs (a) and concomitant ASMs at baseline (b). For bar plots, the x‐axis denotes ASM number/type and the y‐axis denotes the number of patients. ASM, antiseizure medication; CNV, copy number variation; MCD, malformation of cortical development.

Eighty‐seven children with DRE, aged 7–46 months at PER initiation, were included in the safety analysis; 81 who completed at least 3 months of follow‐up were included in the efficacy analysis. Clinical and demographic features are summarized in Table 1 and Figure 2. Baseline analysis indicated that children coadministered oxcarbazepine required significantly higher PER maintenance doses compared with those not receiving oxcarbazepine (3.2 ± 1.3 mg/day vs. 2.5 ± 1.0 mg/day, *p* = 0.022).

**TABLE 1:** Univariate analysis of factors influencing efficacy of adjunctive perampanel in young children with drug‐resistant epilepsy.

Characteristics	Total *n* = 87	3 months OR (95% CI)	*p*	12 months OR (95% CI)	*p*
Sex, female	46 (52.9%)	0.73 (0.29–1.79)	0.492	0.70 (0.29–1.68)	0.427
PER initiation age, months	31.5 [22.0–39.3]	0.99 (0.95–1.04)	0.754	1.01 (0.97–1.06)	0.520
Duration of epilepsy prior to PER, months	16 [8.6–27.0]	0.99 (0.95–1.04)	0.788	1.01 (0.97–1.05)	0.604
Age at seizure onset, months	9 [4.0–18.5]	1.00 (0.96–1.04)	0.985	1.00 (0.96–1.04)	0.945
Family history of epilepsy	15 (17.2%)	1.23 (0.37–3.95)	0.729	1.86 (0.58–6.21)	0.297
Perinatal high‐risk factors	14 (16.1%)	6.43 (1.72–31.27)	0.009	4.73 (1.28–22.82)	0.029
Epileptic seizure types					
Focal seizures	39 (44.8%)	Reference		Reference	
Focal impaired consciousness seizure	14 (16.1%)	Reference		Reference	
Focal preserved consciousness seizure	5 (5.7%)	0.29 (0.01–2.70)	0.324	0.21 (0.01–1.97)	0.217
Focal to bilateral tonic–clonic seizure	20 (23.0%)	0.58 (0.13–2.54)	0.471	1.07 (0.25–4.56)	0.925
Generalized seizures	22 (25.3%)	1.77 (0.58–5.46)	0.313	1.10 (0.37–3.27)	0.862
Generalized tonic–clonic seizure	14 (16.1%)	1.36 (0.29–6.59)	0.695	1.00 (0.21–4.77)	1.000
Generalized myoclonic seizure	5 (5.7%)	0.39 (0.02–4.03)	0.461	0.57 (0.06–4.62)	0.601
Absence seizures	2 (2.3%)	1.17 (0.04–34.03)	0.919	0.86 (0.03–24.95)	0.919
Generalized tonic seizure	1 (1.1%)	> 1000 (< 0.001‐NA)	0.991	> 1000 (< 0.001‐NA)	0.992
Epileptic spasm	26 (29.9%)	0.58 (0.14–2.35)	0.445	0.35 (0.08–1.41)	0.145
DEE	77 (88.5%)	0.59 (0.15–2.31)	0.439	0.49 (0.12–1.86)	0.297
Epileptic syndromes	44 (50.6%)				
IESS	25 (28.7%)	1.11 (0.37–3.26)	0.853	0.62 (0.21–1.75)	0.371
LGS	6 (6.9%)	2.08 (0.34–12.63)	0.408	1.16 (0.19–6.91)	0.867
DS	4 (4.6%)	6.23 (0.72–132.93)	0.128	1.16 (0.13–10.42)	0.889
HHE	3 (3.4%)	2.08 (0.08–55.38)	0.615	1.16 (0.04–30.63)	0.919
EIEE	2 (2.3%)	> 1000 (< 0.001‐NA)	0.995	> 1000 (< 0.001‐NA)	0.995
PME	1 (1.1%)	< 0.001 (NA‐Inf)	0.997	< 0.001 (NA‐Inf)	0.996
MPEI	1 (1.1%)	< 0.001 (NA‐Inf)	0.997	< 0.001 (NA‐Inf)	0.996
DEE‐SWAS	1 (1.1%)	> 1000 (0‐NA)	0.996	> 1000 (0‐NA)	0.996
FIRES	1 (1.1%)				
Etiology					
Genetic	31 (35.6%)	1.35 (0.45–4.17)	0.590	1.36 (0.47–4.05)	0.571
Structural	25 (28.7%)	0.89 (0.27–2.94)	0.846	0.68 (0.21–2.17)	0.515
Autoimmune	2 (2.3%)	1.67 (0.06–45.78)	0.729	1.27 (0.05–34.69)	0.870
CNS infection	2 (2.3%)	< 0.001 (NA‐ > 10^18^)	0.992	1.27 (0.05–34.69)	0.870
Unknown	27 (31.0%)				
Cranial MRI					
Focal	15 (17.2%)	0.74 (0.19–2.68)	0.653	0.36 (0.08–1.31)	0.133
Multifocal	21 (24.1%)	0.79 (0.25–2.40)	0.681	0.65 (0.21–1.96)	0.450
Diffuse	13 (14.9%)	0.24 (0.03–1.07)	0.089	0.27 (0.05–1.06)	0.077
Normal	37 (42.5%)	Reference		Reference	
EEG					
Focal	10 (11.5%)	Reference		Reference	
Multifocal	33 (37.9%)	1.52 (0.34–7.19)	0.584	3.17 (0.70–17.35)	0.148
Extensive	38 (43.7%)	0.45 (0.10–2.11)	0.292	1 (0.22–5.33)	1
Normal	2 (2.3%)	< 0.001 (NA‐ > 10^18^)	0.992	2 (0.06–64.48)	0.661
EEG background slow activity	34 (39.1%)	0.71 (0.28–1.77)	0.470	0.89 (0.37–2.17)	0.802
Comorbidities					
Global developmental delay	77 (88.5%)	0.60 (0.15–2.36)	0.455	0.47 (0.11–1.82)	0.281
Autism spectrum disorder	1 (1.1%)	> 1000 (0‐NA)	0.996	> 1000 (< 0.001–NA)	0.995
Status epilepticus	27 (31.0%)	1.75 (0.67–4.62)	0.254	2.50 (0.96–6.74)	0.063
Number of previous ASMs	4 [3–6]	0.50 (0.11–2.02)	0.337	0.60 (0.15–2.34)	0.465
Number of concomitant ASMs	2 [2–3]	0.07 (0–0.50)	0.022	0.09 (0–0.56)	0.030
Prior ketogenic diet	26 (29.9%)	0.41 (0.13–1.15)	0.104	0.40 (0.14–1.07)	0.077
Concomitant ketogenic diet	10 (11.5%)	1.69 (0.43–6.63)	0.439	1.29 (0.33–5.02)	0.706
Prior VNS	2 (2.3%)	< 0.001 (NA‐ > 10^18^)	0.992	< 0.001 (NA‐ > 10^18^)	0.992
Concomitant VNS	4 (4.6%)	< 0.001 (NA‐ > 10^18^)	0.991	< 0.001 (NA‐ > 10^18^)	0.991
Prior steroid use	31 (35.6%)	0.50 (0.18–1.32)	0.174	0.36 (0.13–0.93)	0.040
Concomitant steroid use	13 (14.9%)	0.42 (0.09–1.51)	0.215	0.32 (0.07–1.15)	0.103
Initial dose of PER, mg/day	0.5 [0.5–1]	0.92 (0.50–1.63)	0.782	0.91 (0.51–1.59)	0.749
Maintenance dose of PER, mg/day	2 [2–4]	0.84 (0.54–1.29)	0.421	0.78 (0.51–1.19)	0.259

*Note:* Continuous variables are expressed as median [interquartile range]; categorical variables are given as *n* (%). Missing data: 1 case for PER initiation age, 6 cases for age at seizure onset, 6 cases for duration of epilepsy before PER, 1 case for cranial MRI, and 4 cases for EEG.

Abbreviations: ASMs, antiseizure medications; CI, confidence interval; CNS, central nervous system; DEE, developmental and epileptic encephalopathy; DEE‐SWAS, developmental and epileptic encephalopathy with spike‐and‐wave activation in sleep; EEG, electroencephalogram; EIEE, early infantile epileptic encephalopathy; FIRES, febrile infection‐related epilepsy syndrome; HHE, hemiconvulsion–hemiplegia epilepsy; IESS, infantile epileptic spasms syndrome; LGS, Lennox–Gastaut syndrome; DS, Dravet syndrome; MPEI, migrating partial epilepsy of infancy; MRI, magnetic resonance imaging; OR, odds ratio; PER, perampanel; PME, progressive myoclonus epilepsy; VNS, vagus nerve stimulation.

### 3.2 Efficacy Outcomes

**FIGURE 3:**
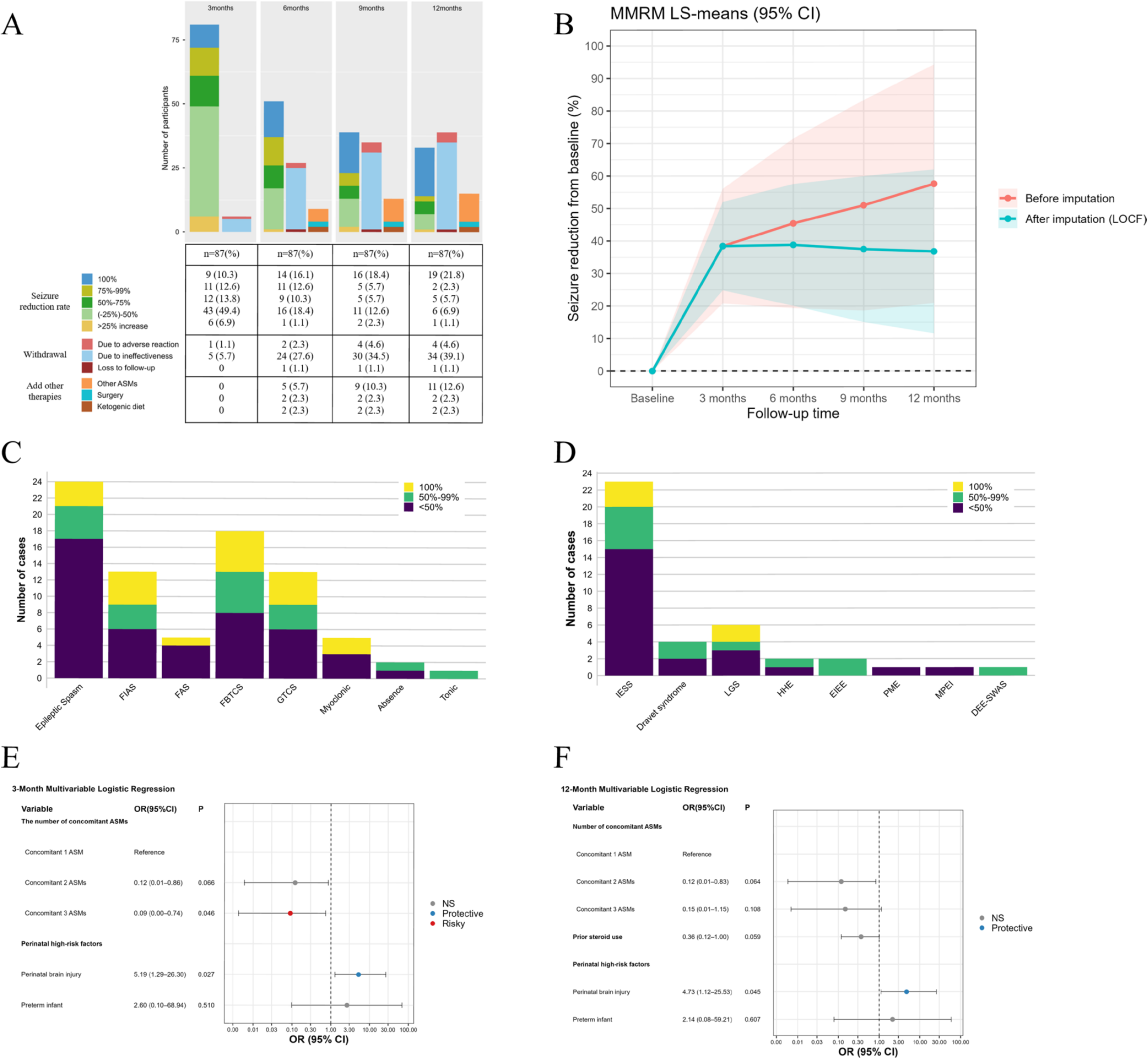
Efficacy outcomes of adjunctive perampanel. (A) Responder and seizure‐freedom rates at 3, 6, 9, and 12 months. (B) Mean percent reduction in seizure frequency from baseline using MMRM with a Toeplitz covariance structure, with LOCF sensitivity analysis. (C) Responder rates by seizure type at 12 months. (D) Responder rates by epilepsy syndrome at 12 months. For bar plots, the x‐axis denotes seizure type or syndrome and the y‐axis the responder rate (%). (E, F) Multivariable logistic regression of predictors of treatment response at 3 and 12 months. FBTCS, focal‐to‐bilateral tonic–clonic seizures; GTCS, generalized tonic–clonic seizures; FIAS, focal impaired awareness seizures; FAS, focal aware seizures; IESS, infantile epileptic spasms syndrome; DS, Dravet syndrome; LGS, Lennox–Gastaut syndrome; LOCF, last observation carried forward; MMRM, mixed model for repeated measures; OR, odds ratio.

To limit potential upward bias in seizure‐freedom and responder rates arising from loss to follow‐up and postbaseline treatment modifications, we conducted sensitivity analyses using LOCF imputation and MMRM. In the MMRM (Toeplitz covariance), adjunctive PER reduced mean seizure frequency by 38.5%, 40.6%, 42.2%, and 46.7% at 3, 6, 9, and 12 months, respectively (*p* < 0.001 for all comparisons vs. baseline), indicating a significant and durable treatment effect (Figure 3A,B). Sensitivity analyses comparing estimates before and after LOCF imputation showed no statistically significant differences at any visit (3 months: *p* = 0.927; 6 months: *p* = 0.952; 9 months: *p* = 0.823; 12 months: *p* = 0.639), suggesting that LOCF did not materially affect the primary conclusions. In the imputed dataset, PER significantly reduced seizure frequency over the 12 month follow‐up. Responder rates were 39.5% (32/81), 46.9% (38/81), 43.2% (35/81), and 44.4% (36/81) at 3, 6, 9, and 12 months, respectively. Seizure‐freedom was achieved in 12.3% (10/81), 19.8% (16/81), 19.8% (16/81), and 23.5% (19/81) of patients at these time points.

At 12 months, responder rates varied across seizure types and epilepsy syndromes (Figure 3). Responder rates (≥ 50% reduction from baseline) were 55.6% (10/18) for focal‐to‐bilateral tonic–clonic seizure (FBTC), 53.9% (7/13) for generalized tonic–clonic seizure (GTC), 53.9% (7/13) for focal impaired consciousness seizure (FIC), 40.0% (2/5) for myoclonic seizure, 29.2% (7/24) for epileptic spasms, 20.0% (1/5) for focal preserved consciousness seizure (FPC), and 50.0% (1/2) for absence seizures. Among syndromic diagnoses, responder rates were 50.0% (2/4) for DS, 50.0% (3/6) for LGS, and 34.8% (8/23) for infantile epileptic spasms syndrome (IESS). Children with genetic etiologies had a responder rate of 51.7% (15/29), including 60.0% (3/5) in those with *SCN1A* variants.

Using ≥ 50% seizure reduction at 3 and 12 months as the dependent outcomes, univariable logistic regression identified several baseline variables with potential influence on treatment response (Table 1). Multivariable analysis demonstrated that concomitant use of three ASMs (OR = 0.09, 95% CI < 0.01–0.74) predicted reduced odds of achieving ≥ 50% seizure reduction at 3 months. Perinatal brain injury independently predicted favorable response at both 3 months (OR = 5.19, 95% CI 1.29–26.30) and 12 months (OR = 4.73, 95% CI 1.12–25.53) (Figure 3E,F).

### 3.3 Treatment Retention and Safety

**FIGURE 4:**
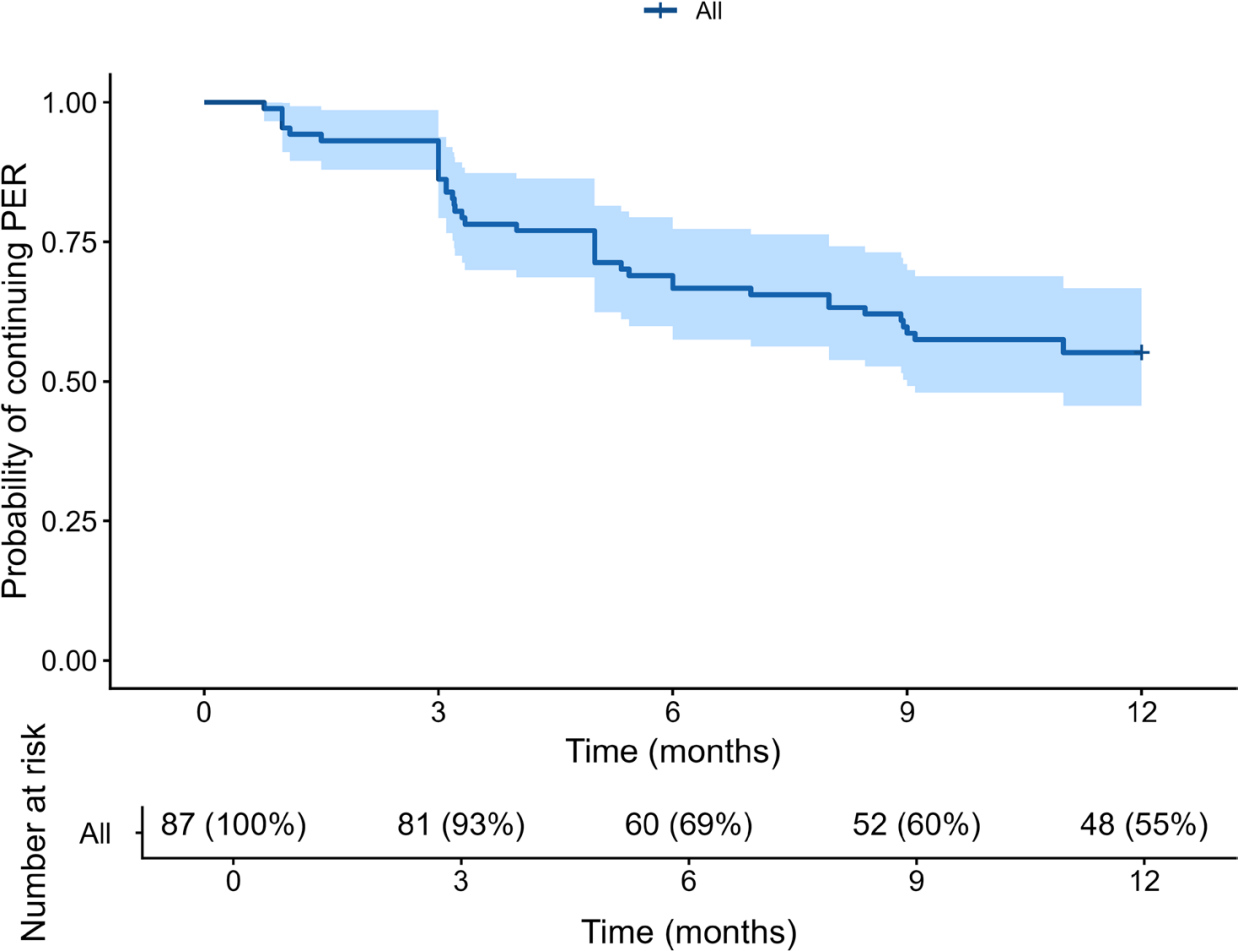
Treatment retention with adjunctive perampanel. Kaplan–Meier curve showing retention up to 12 months, with 95% confidence intervals. The risk table shows the number of patients at risk at each time point.

Median treatment exposure was 12.0 months (range 0.8–12.0). The Kaplan–Meier estimate of treatment retention was 87.4% at 3 months, declining to 55.2% at 12 months (Figure 4). By the end of the 12 month follow‐up, 39 children (44.8%) had not remained on PER treatment. The specific reasons included insufficient efficacy in 32 children (36.8%), parental request for medication change due to long‐term seizure control not meeting expectations in 2 children (2.3%), discontinuation due to TEAEs in 4 children (4.6%), and loss to follow‐up in 1 child (1.1%). Median time to discontinuation was 4.0 months (IQR 2.1–7.0).

TEAEs were reported in 20 of 87 patients (23.0%). The most frequent events were somnolence (11.5%) and irritability or aggressive behavior (9.2%). Ataxia occurred in 4.6%, and headache, dizziness, fatigue, and decreased appetite each in ≤ 2.3%. Symptoms were alleviated with dose reduction or supportive management in the 16 patients (18.4%) who remained on PER treatment. No serious or unexpected safety signals were observed.

## 4 Discussion

Epilepsy is a common neurological disorder across all age groups, with infants and older adults at particularly high risk [15]. Among children under 5 years, the global prevalence is estimated at 0.29%, while in China it is 0.22% with an annual incidence of 45.24 per 100,000 [16]. Evidence for the use of PER in children younger than 4 years with DRE remains limited. Real‐world evidence, by reflecting outcomes in routine clinical practice, complements trial data and can inform individualized care. In this study, we evaluated the efficacy and safety of adjunctive PER in young children with DRE. Overall, PER was effective and generally well tolerated, providing valuable data to guide treatment selection in this challenging age group.

### 4.1 Efficacy

Previous studies have suggested that adjunctive PER yields responder rates of 33.3%–64.5% and seizure‐freedom rates of 10.0%–17.9% in children younger than 4 years [8, 9, 10]. In our cohort, the 12 month responder and seizure‐freedom rates were 44.4% and 23.5%, respectively, slightly lower than in our earlier study of children aged 4–12 years [17]. The present population had more severe baseline disease, with 88.5% meeting criteria for developmental and epileptic encephalopathy (DEE) and a higher median number of previously tried ASMs.

PER is recommended as first‐line therapy for focal seizures (FS), and both monotherapy and adjunctive therapy have demonstrated efficacy in FS and FBTC [18, 19, 20, 21]. PER is also effective against GTC and may be considered for myoclonic and absence seizures [22, 23, 24, 25]. In this cohort, after inadequate response to first‐line antiseizure therapies, PER was administered to children with GTC, FS, epileptic spasms, and generalized myoclonic seizures. PER is also recommended for several epilepsy syndromes, including DS, LGS, and childhood absence epilepsy (CAE) [22, 26, 27, 28, 29, 30, 31]. In our syndrome‐specific analyses, responder rates were 50.0% for DS, 50.0% for LGS, and 34.8% for IESS, supporting PER as an adjunctive therapy for DEEs in young children.

Childhood‐onset epilepsies are etiologically diverse: approximately 30% are genetic or presumed genetic overall, rising to approximately 80% in infantile‐onset cases [32, 33]. In genetic epilepsies, impaired GABAergic inhibition or pyramidal neuron hyperexcitability can drive glutamatergic overactivation, making AMPA receptor antagonism a rational approach [26]. Previous studies have reported good efficacy of PER in *SCN1A*‐, *GNAO1*‐, *PIGA*‐, *PCDH19*‐, *SYNGAP1*‐, *CDKL5*‐, *NEU1*‐, and *POLG*‐related epilepsies [26]. In our study, 35.6% of children had genetic etiologies, with a responder rate of 51.7% overall and 60.0% in those with *SCN1A* variants.

Treatment response was also influenced by baseline therapy. In the PERaMpanel pooled analysis of effectiveness and tolerability (PERMIT) study, fewer previously used and fewer concomitant ASMs at baseline predicted better outcomes [34]. In our cohort, the number of prior ASMs was not independently associated with efficacy, but concomitant use of three ASMs significantly reduced the odds of response, consistent with prior reports [17]. Notably, because all patients had received at least two ASMs before initiation of PER, the range of the “number of prior ASMs” variable was limited, constraining its interpretability. Additionally, children receiving oxcarbazepine required higher PER maintenance doses, suggesting that when PER is coadministered with enzyme‐inducing ASMs (EIASMs), a faster titration and a higher maintenance dose may be warranted. PER displays linear, first‐order pharmacokinetics with dose‐proportional increases in plasma concentration, and routine therapeutic drug monitoring (TDM) is generally unnecessary. Pediatric pharmacokinetics typically differ from adult profiles, with faster drug clearance in children. Because PER is primarily metabolized by CYP3A4, coadministration with CYP3A4 inducers or inhibitors can substantially alter exposure and clinical response. With oxcarbazepine, the area under the concentration–time curve decreases by approximately 48% and clearance increases by about 84.1% [3]. Pharmacogenetic polymorphisms have been shown to influence PER plasma concentrations in Chinese children [35, 36]. Accordingly, in young Chinese patients harboring CYP3A5*3 or CYP3A4*10 variants, in those receiving EIASMs, or when severe adverse events occur, TDM may be considered on a case‐by‐case basis, taking institutional capacity into account.

Children with perinatal risk factors, mainly perinatal hypoxic–ischemic brain injury, appeared to derive greater benefit from PER treatment. The underlying mechanism may be closely related to glutamate‐mediated excitotoxicity and AMPA receptor–dependent network hyperexcitability induced by hypoxic–ischemic brain injury. Previous experimental studies have shown that neonatal hypoxic–ischemic injury and hypoxia‐induced seizures can alter AMPAR subunit composition and receptor trafficking, characterized by downregulation of the GluA2 subunit, increased calcium‐permeable AMPAR activity, and enhanced GluA1 phosphorylation, thereby promoting persistent excitatory synaptic remodeling and epileptogenesis [37]. As a highly selective, noncompetitive AMPA receptor antagonist, PER can directly inhibit excitatory transmission mediated by calcium‐permeable AMPA receptors [37]. Moreover, animal hypoxic–ischemic models and clinical studies of post‐cardiac arrest encephalopathy have shown that PER may exert neuroprotective effects through this target [38, 39]. These mechanisms suggest that AMPA receptors may play an important pathogenic role in structural epilepsies represented by perinatal brain injury, making targeted intervention with PER more biologically relevant.

### 4.2 Retention and Safety

Treatment retention integrates efficacy and tolerability over time. In this study, 12 month retention was 55.2%, closely aligned with the 50% reported in the PROVE cohort [7], supporting PER as a viable long‐term option in very young children.

Discontinuations were driven primarily by TEAEs and inadequate seizure control [40]. In the open‐label Study 311 (aged 4–12 years), the most frequent TEAE was somnolence (26%), and TEAE‐related discontinuation occurred in 4%–11% [41]. In the PROVE pediatric analysis, TEAE incidence was 29.3% in children < 4 years and 31.5% in those 4–12 years. The most common events were irritability (7.3%) and aggression (6.9%), and TEAE‐related discontinuation within 24 months was < 21% [7]. In our cohort, TEAEs occurred in 23.0% of patients, with somnolence and irritability/aggressive behavior being most common. Only 4.6% discontinued due to TEAEs, consistent with earlier pediatric and adult reports [40, 41]. Most events were mild to moderate and manageable with slower titration, lower starting doses, or dose adjustment. The overall safety profile in this very young cohort was comparable to that seen in older children, characterized mainly by manageable, mild‐to‐moderate neuropsychiatric symptoms, underscoring the feasibility of PER in young children with DRE.

This real‐world study indicates that adjunctive PER confers meaningful efficacy with a favorable safety profile in young children with DRE (7–46 months of age). Benefits were particularly notable in those with GTC or perinatal brain injury. TEAEs were predominantly mild to moderate and generally controllable, supporting PER as a therapeutic option in this population.

This study also has several limitations, including a relatively modest sample size, limited follow‐up duration, and insufficient statistical power for some subgroup analyses. In particular, the efficacy data for rare epilepsy syndromes, such as DS (*n* = 4) and LGS (*n* = 6), should be interpreted as exploratory findings based on small samples and have not reached confirmatory statistical power. Therefore, these findings should not be taken as sufficient evidence to support the first‐line use of PER in these specific populations. Further larger‐scale prospective studies are needed to validate the efficacy and safety of PER in this population, optimize individualized treatment strategies, and provide more robust scientific evidence for precision therapy in young children with DRE.

## Author Contributions

Qiao Zeng, Xueqian Xia, and Yue Hu contributed to the conceptualization and design of the study, data acquisition and analysis, and drafting of the manuscript. All authors contributed to the data acquisition, review, and editing.

## Ethics Statement

This study was conducted in accordance with the Declaration of Helsinki and was approved by the Ethics Committee of Children's Hospital of Chongqing Medical University (346).

## Consent

Informed consent was obtained from the parents or legal guardians of all participants included in the study.

## Conflicts of Interest

The authors declare no conflicts of interest.

## Data Availability

The data that support the findings of this study are available from the corresponding author upon reasonable request.
